# An Atypical Flavin-Containing Monooxygenase Homologue Catalyzes the Third Epoxidation in the Verrucosidin Biosynthesis

**DOI:** 10.1021/acs.orglett.6c02534

**Published:** 2026-07-04

**Authors:** Hui-Ling Wei, Xiao-Ling Chen, Wen-Bing Yin, Jie Fan, Shu-Ming Li

**Affiliations:** † 9377 Philipps-Universität Marburg, Fachbereich Pharmazie, Institut für Pharmazeutische Biologie und Biotechnologie, Robert-Koch-Straße 4, 35037 Marburg, Germany; ‡ State Key Laboratory of Mycology, 85387 Institute of Microbiology, Chinese Academy of Sciences, Beijing, 100101, P. R. China; § Department of Microbiology, College of Life Science, 12538 Nankai University, Tianjin, 300071, P. R. China

## Abstract

*Penicillium polonicum* CGMCC 3.15272 converts deoxyverrucosidin to verrucosidin by epoxidation with an atypical 805-amino acid large flavin-dependent monooxygenase homologue, VecG. Gene deletion, heterologous expression, and mutagenesis proved that both its predicted 458-amino acid N-terminal FMO core and the 242-amino acid C-terminal transmembrane region are essential for the catalysis. VecG differs from its inactive orthologue DovG in strain NRRL 995 in only three amino acids, two of which are more critical for the activity.

## Graphical abstract

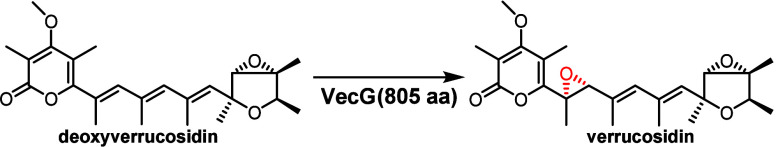

Scaffold modification, including late-stage oxidative tailoring, enables biosynthetic pathways to generate structurally distinct products from closely related intermediates.^1,2^ Usually, even a single oxygenation event can alter product structure. Flavin-dependent monooxygenases (FMOs) often play important roles in these processes by epoxidation or hydroxylation.^3–7^ This is particularly relevant for pathways that produce closely related metabolites differing only in oxidation state.

Verrucosidin and deoxyverrucosidin provide an excellent example for addressing this phenomenon. The two fungal polyketides differ solely by an additional epoxide ring on the polyene chain of verrucosidin,^8^ suggesting that they share early biosynthetic steps and diverge at the final epoxidation step. In our previous work, we identified deoxyverrucosidin as the final product of the Dov pathway in *P. polonicum* NRRL 995 and assigned DovC (FMO), DovD (epoxide expandase), and DovE (P450 enzyme) to the first epoxidation, epoxide expansion to a dihydrofuran ring, and second epoxidation (Figure 1).^9^ In other *P. polonicum* strains, however, verrucosidin and other oxidized deoxyverrucosidin derivatives have been reported.^8,10–12^ Furthermore, the verrucosidin production has been linked to a biosynthetic gene cluster (*ver*) in *P. polonicum* X6, without detailed characterization.^13^ These facts prompted us to identify the enzyme(s) responsible for the third epoxidation in the verrucosidin biosynthesis, i.e., the conversion of deoxyverrucosidin to verrucosidin.

**Figure 1:**
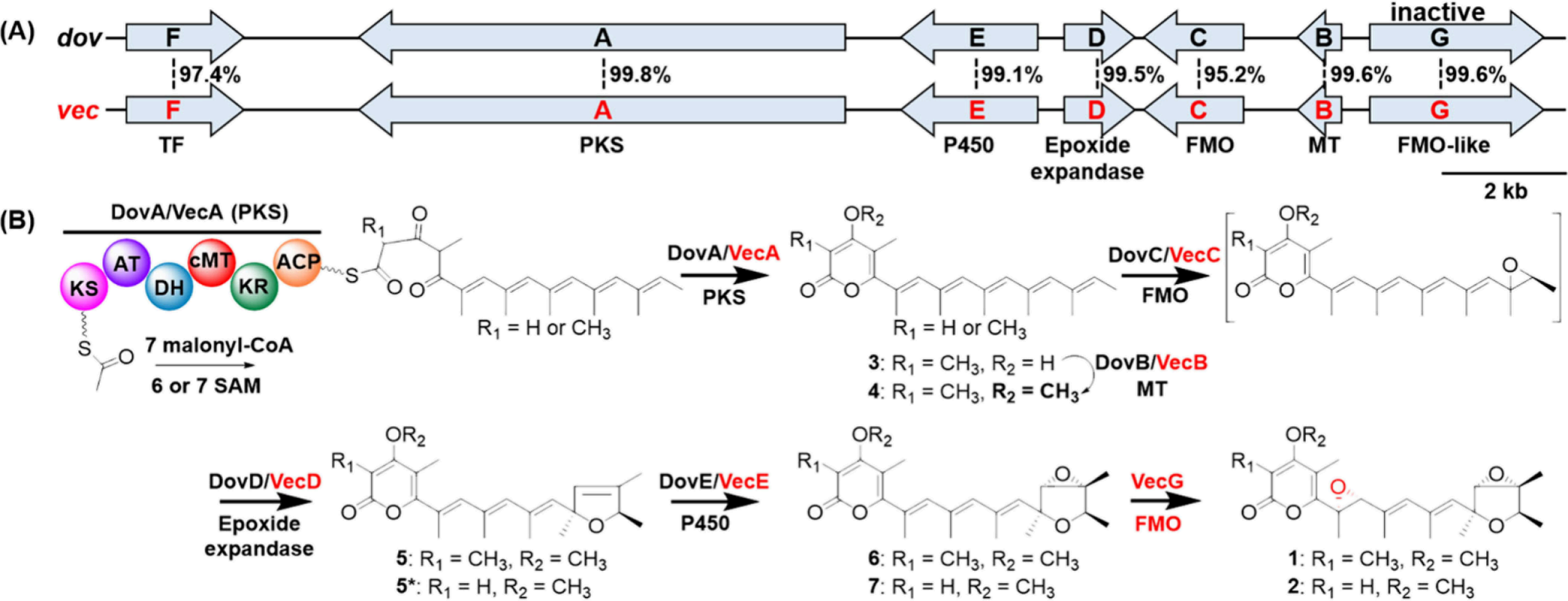
(A) The *dov* cluster in *P. polonicum* NRRL 995 and *vec* cluster in *P. polonicum* CGMCC 3.15272. (B) Proposed biosynthetic pathway for verrucosidin (**1**) and congeners. 5* was not detected in the strains. PKS, polyketide synthase; MT, methyltransferase; FMO, flavin-dependent monooxygenase; P450, Cytochrome P450; and TF, transcription factor.

Guided by the previously characterized *dov* cluster, we identified a highly homologous locus (*vec*) in *P. polonicum* CGMCC 3.15272 and linked it to the production of verrucosidin. We demonstrated that the atypical large FMO homologue VecG catalyzes the third epoxidation in verrucosidin biosynthesis. Both the predicted FMO catalytic core and the transmembrane region are required for activity. VecG differs from its inactive orthologue DovG in only three amino acids, two of which are important for the activity.

Detailed analysis revealed that the *vec* and *dov* clusters share more than 95% sequence identity on the amino acid level and the same gene organization (Figure 1 and Table S4). Besides genes for polyketide assembly, the *vec* locus contains the same set of oxidative tailoring enzymes as the *dov* cluster, including two FMOs VecC and VecG, an epoxide expandase VecD, and a cytochrome P450 VecE. The smaller 467-amino acid FMO VecC corresponds to the first epoxidase DovC, whereas the larger 805-amino acid FMO VecG is the homologue of DovG, which was proven not involved in the deoxyverrucosidin biosynthesis.^9^ This high degree of sequence conservation suggests the same biosynthetic framework and similar or even the same reaction steps of the two pathways. Therefore, it is important to analyze the metabolite profile of CGMCC 3.15272 and identify the final pathway product of the *vec* cluster.

For this purpose, both *P. polonicum* NRRL 995 and CGMCC 3.15272 were cultivated on rice medium for 14 days under identical conditions. LC-MS analysis of the ethyl acetate extracts revealed highly similar metabolite profiles in the two strains, with most metabolites displaying comparable retention times, peak shapes, and UV absorption profiles (Figure S1). Peaks matching deoxyverrucosidin and nordeoxyverrucosidin were also detected in CGMCC 3.15272 at low levels, while two additional peaks were observed exclusively in this strain (Figures 2 and S1). The major peak **1** eluted approximately 1 min earlier than deoxyverrucosidin and showed an [M + H]^+^ ion at *m*/*z* 417.2295, which is 16 Da larger than that of deoxyverrucosidin, indicating a monooxygenation. Compared with deoxyverrucosidin, **1** showed a hypsochromic shift in the UV maximum from 332 to 296 nm, suggesting an interruption of the conjugated system. The second peak **2** gave an [M + H]^+^ ion at *m*/*z* 403.2128, corresponding to a demethylated analogue of **1**, as in the case of deoxyverrucosidin and nordeoxyverrucosidin. Together, these data suggested the presence of further oxidized congeners of deoxyverrucosidin.

**Figure 2:**
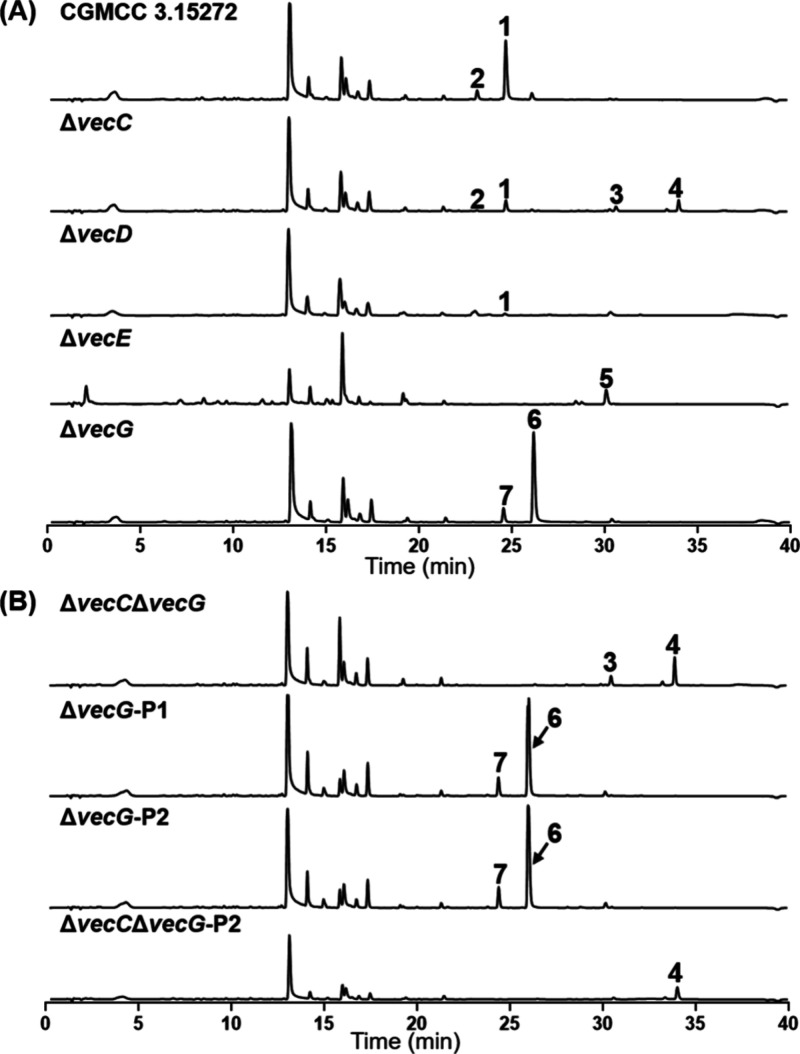
HPLC profiles of the metabolites from *P. polonicum* strains. The chromatograms were monitored at 280 nm.

Compounds **1** and **2** were purified by HPLC and identified by LC-MS and NMR as verrucosidin and normethylverrucosidin, respectively (Figures S2–S4 and Table S5). Both were identified in *Penicillium verrucosum* var. *cyclopium.* ^14–16^ Obviously, strain CGMCC 3.15272 can convert deoxyverrucosidin to verrucosidin as the final product through an additional epoxidation. However, it cannot be excluded that an enzyme encoded by a gene outside of the *vec* cluster catalyzes the final epoxidation step.

To determine the oxidative tailoring steps in the Vec pathway, we individually deleted four candidate genes, *vecC*, *vecD*, *vecE*, and *vecG*, in CGMCC 3.15272 (Figure S5). Disruption of *vecD* markedly reduced the yields of **1** and **2** and led to the accumulation of hydrolysis products, consistent with the *dovD* deletion mutant in NRRL 995 (Figure 2A).^9^ In the deoxyverrucosidin pathway, DovC shows limited ability to form the 2,5-dihydrofuran intermediate, whereas DovD enables efficient and stereocontrolled conversion.^9^ Similarly, the residual production of **1** in the Δ*vecD* mutant may arise from the low level dihydrofuran intermediate produced by VecC, supporting the function of VecD as the epoxide expandase. Likewise, disruption of *vecE* coding for a P450 enzyme abolished **1** and **2** production and led instead to accumulation of compound **5**, a precursor containing the 2,5-dihydrofuran ring (Figure 2A), confirming its role in the second epoxidation step (Figure 1B).

A different metabolic outcome from that of the *dov* cluster was observed for the two FMO deletion mutants. Deletion of *vecG* abolished **1** and **2** production and resulted in deoxyverrucosidin (**6**) and nordeoxyverrucosidin (**7**) accumulation, which were identified by comparison of retention times, MS, and UV data with those of authentic standards and also by NMR for **6**. This result proved unequivocally the requirement of the unusual 805-amino acid large FMO VecG for the third epoxidation and thus for the final conversion to verrucosidin-type products (Figure 1B).

Deletion of *dovC* in NRRL 995 resulted in the accumulation of the polyketide **3** and its *O*-methylated derivative **4**.^4^ In comparison, compounds **3** and **4** were also accumulated in the Δ*vecC* mutant, but with very low product yields. In addition, compounds **1** and **2** are still detected, although with a markedly reduced level compared to the wild type (Figure 2A). This indicates that VecC has a major contribution to pathway flux during early stage epoxidation, but is not strictly required for the formation of verrucosidin-type products. Another enzyme in the verrucosidin pathway or outside the cluster can complement, at least partially, the function of VecC.

Based on the metabolite profiles of Δ*vecC*, Δ*vecD*, Δ*vecE*, and Δ*vecG* mutants, we proposed a biosynthetic pathway for verrucosidin, which shares the same early steps to deoxyverrucosidin with the Dov pathway in NRRL 995 (Figure 1B). It begins with the formation of the PKS product **3** by VecA, followed by *O*-methylation with VecB to give **4**. As in the deoxyverrucosidin biosynthesis, VecC catalyzes the first epoxidation, VecD mediates epoxide expansion, and the P450 enzyme VecE catalyzes the second epoxidation of the 2,5-dihydrofuran-containing intermediate **5**. Differing from the Dov pathway, the resulting intermediates **6** and **7** are then converted by the atypically large FMO homologue VecG as the third epoxidase, yielding **1** and **2**, respectively (Figure 1B).

As mentioned above, the low-level production of **1** and **2** in the Δ*vecC* mutant suggested that the first epoxidation by VecC was partially complemented by another enzyme. We proposed that VecG, which also catalyzes epoxidation of the polyene chain, was a possible candidate. To prove this hypothesis, we constructed a Δ*vecC*Δ*vecG* mutant (Figure S5) and analyzed its metabolite profile. As shown in Figure 2B, no epoxidized metabolites such as **1**, **2**, **5**, **6**, and **7** were detected. Instead, **3** and its methylated precursor **4** were accumulated as the minor and major products, respectively. This proved that the accumulated verrucosidin-type metabolites in the Δ*vecC* mutant were a result of the VecC function replacement by the promiscuous VecG, rather than by another enzyme outside the cluster. In contrast, VecG function cannot be complemented by VecC, as demonstrated in the Δ*vecG* mutant (Figure 2A).

The different substrate flexibilities of VecG and VecC prompted us to examine whether the unusual size of VecG is functionally relevant. Sequence analysis showed that the 805-aa large VecG contains a predicted 458-aa N-terminal FMO core, comparable in size to typical FMOs like VecC (467 aa), and a predicted 242-aa C-terminal transmembrane region (Figure S6). To assess the importance of both regions, truncated variants of VecG were generated (Figure S5). In the Δ*vecG*-P1 mutant, the region encoding amino acid residues 1–471 containing the predicted FMO core was deleted, whereas Δ*vecG*-P2 lacks residues 525–805 in the predicted transmembrane region of *vecG*. LC-MS analysis revealed the abolishment of **1** and **2** production in both Δ*vecG*-P1 and Δ*vecG*-P2, and instead accumulation of **6** and **7**, demonstrating that both regions in VecG are required for the third oxidation (Figure 2B). To determine whether the remaining FMO part of VecG could complete the first VecC-catalyzed epoxidation, *vecC* was additionally deleted in the Δ*vecG*-P2 mutant. The resulting mutant accumulated precursor **4** and failed to produce any epoxidized metabolites (Figure 2B), indicating that the predicted transmembrane region of VecG is also essential for its catalytic activity toward **4**.

Most functionally characterized FMOs in fungal natural product biosynthesis are conventional-sized enzymes (typically 450–500 aa), as exemplified by AurC,^17^ CtvC,^18^ and AstC,^19^ which mainly catalyze iterative epoxidation and related oxidative transformations in related polyene pyrone pathways. Notably, AurC has been described as a predicted membrane-anchored monooxygenase.^17^ Slightly larger or domain-extended FMOs are known in other biological systems. For example, the 558-aa large bacterial monooxygenase OnpA contains a fused cytochrome *b*5 domain, which is required for the activity.^20^ However, VecG with 805 aa is much larger than known FMO enzymes with additional domains.

Searching the Swiss-Prot database using VecG as a query identified 42 functionally characterized or proposed FMOs for epoxidation. Of these, 36 are conventional-sized enzymes (<500 aa), three are 500–700 aa, and only three exceed 700 aa. Phylogenetic analysis showed that the proteins larger than 500 aa are closely located together. VecG is located within a clade containing members of >700 aa, including AtnA, AtnK, and NtnK from a meroterpenoid biosynthetic pathway^21^ (Figure S7). These three proteins share a VecG-like architecture with an FMO-like and an N-terminal transmembrane region (Figure S6). AtnA has been linked to an epoxidation step by gene deletion experiment without detailed characterization, whereas AtnK and NtnK were proposed as epoxidases based on pathway logic and comparative bioinformatics.^21^ However, the domain organization of these proteins was not discussed in the original study, and the functional contribution of the predicted membrane-associated region remains unknown.

Database search identified at least 76 additional uncharacterized VecG-related proteins in diverse fungal genomes, with >90% query coverage and >40% sequence identity to VecG (Table S6). These proteins are mostly annotated as hypothetical, uncharacterized, or FAD/NAD­(P)-binding domain-containing proteins without experimental validation. Together with the predicted similarity in size and domain organization, this analysis suggests an until now almost unexplored group of large FMO-like proteins. Their metabolic functions, whether as epoxidases or other types of biocatalysts, and the functional contribution of the predicted membrane domain remain to be elucidated.

Having shown that both the predicted FMO core and the transmembrane region are required for VecG function, we next examined the difference between VecG and its closely related homologue DovG, which had previously been shown to be inactive in the deoxyverrucosidin pathway. Initial attempts to characterize VecG in *E. coli* using crude protein extract or feeding experiments were unsuccessful. We therefore expressed *vecG* and *dovG* in *A. nidulans* LO8030 under the constitutive *gpdA* promoter.^22^ Feeding of **6** and **7** to the resulting strains showed that VecG converted them almost completely to **1** and **2**, respectively, whereas DovG showed no detectable conversion (Figure 3A), confirming its inactivity in the deoxyverrucosidin pathway as previously reported.^9^

**Figure 3:**
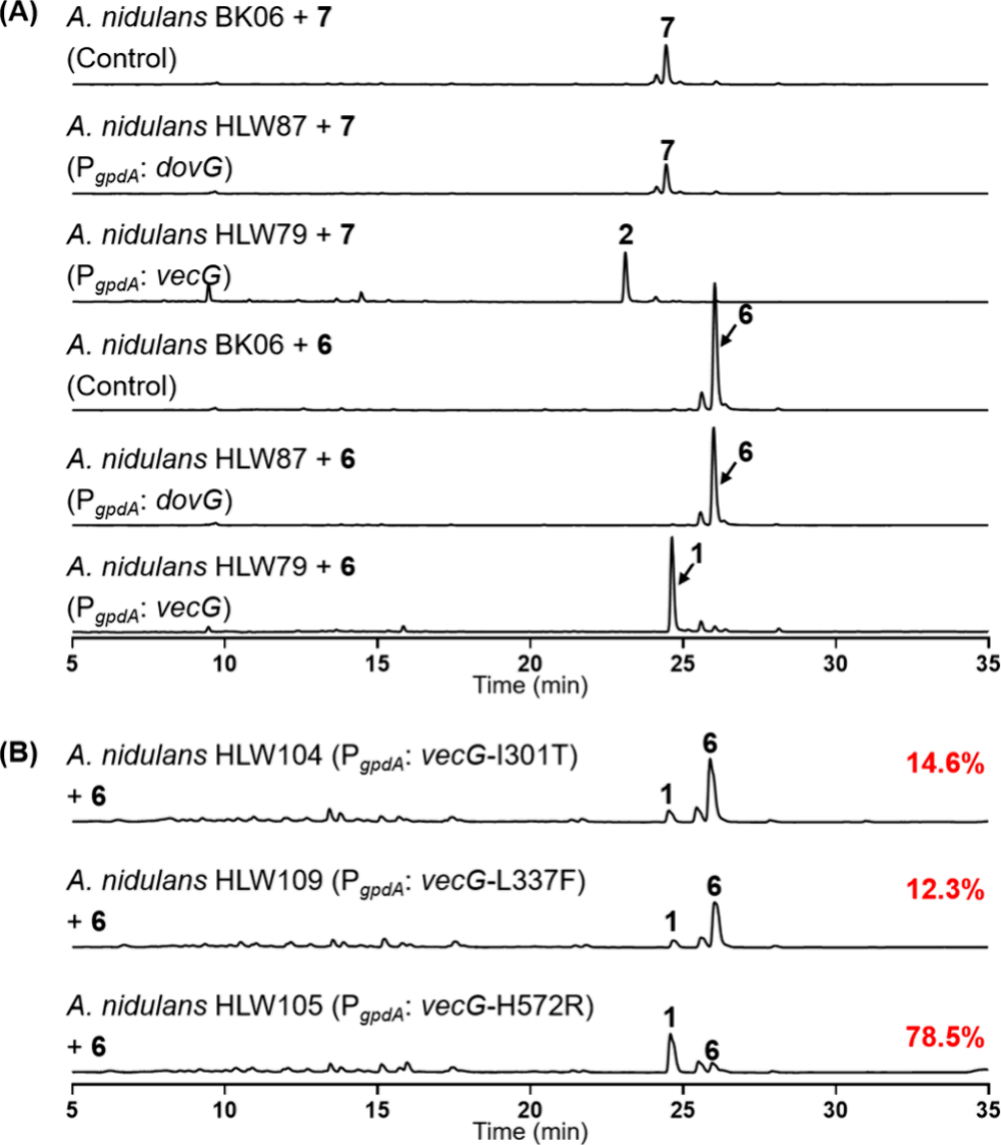
HPLC profiles of the metabolites from *P. polonicum* strains. The chromatograms were monitored at 280 nm.

Multiple-sequence alignments of VecG, DovG, and related large-FMO homologues from *P. polonicum* strains showed high conservation across the full-length proteins, including both the predicted catalytic region and the transmembrane segment (Figure S8). Sequence variation between VecG and DovG is restricted to only three amino acid residues, i.e., positions 301 and 337 in the FMO core and position 572 in the transmembrane region. At position 301, VecG contains Ile, whereas DovG contains Thr, representing a hydrophobic-to-polar substitution. At position 337, VecG contains Leu, whereas DovG contains Phe, corresponding to an aliphatic to aromatic change. At position 572, VecG contains His, whereas DovG contains Arg, representing a substitution between two basic residues with distinct side chain properties. Most of the other homologues retain the same residues as those of VecG at these positions, suggesting that the functional difference between VecG and DovG is likely associated with these residues.

Single substitutions were introduced at the three positions in VecG, and the resulting constructs were expressed in *A. nidulans* LO8030. The overexpression strains were individually fed with **6**. As shown in Figure 3B, significantly reduced product formation was observed for the I301T and L337F mutants, with conversion yields of 14.6% and 12.3%, respectively, whereas only slightly lower activity was detected for H572R, with a conversion yield of 78.5%. In contrast, complete conversion was observed for the nonmutated control strain under the same conditions (Figure 3A). These results indicate that residues 301 and 337 in the FMO core are more important than residue 572 for the VecG activity.

AlphaFold-based structural prediction for VecC, VecG, and DovG indicated very similar domain architectures of the FMO core sequences, even for the three different amino acids in VecG and DovG. It seems that these residues contribute to the catalytic activity, but not the protein fold. The C-terminal sequences in VecG and DovG appear as extended α-helices (Figure S9). VecG acts on a highly hydrophobic epoxytetrahydrofuran intermediate constructed by three membrane-bound enzymes VecC, VecD, and VecE. The extra membrane region in VecG could help it to position in such an environment for effective epoxidation.

In summary, we identified the *vec* cluster in *P. polonicum* CGMCC 3.15272 and proved its role in verrucosidin biosynthesis, involving three sequential epoxidation steps catalyzed by three different enzyme types. Although highly similar to the previously characterized *dov* cluster, the *vec* cluster produces verrucosidin with one additional epoxide on the polyene chain. This feature is introduced by the active atypical large FMO homologue VecG, whereas its orthologue DovG from the deoxyverrucosidin producer NRRL 995 was proven to be inactive. A BLASTP search revealed numerous VerG/DovG homologues of similar size containing both an FMO catalytic core and a transmembrane region, indicating the presence of a widely distributed but poorly characterized group of large FMO-like enzymes. Elucidation of their functions in natural-product biosynthesis will be an interesting topic for future research.

## Supplementary Material



## Data Availability

The data underlying this study are available in the published article and its Supporting Information.

## Notes

Additional experimental details, materials and methods, including strains, physiochemical properties, LC-MS, and NMR data (PDF)
