# Supplementary material for: An Atypical Flavin-Containing Monooxygenase Homologue Catalyzes the Third Epoxidation in the Verrucosidin Biosynthesis
Source: Org Lett. 2026 Jul 4;28(28):9048–52. doi: 10.1021/acs.orglett.6c02534 (PMC13386531; doi:10.1021/acs.orglett.6c02534)
Supplement: Supplementary file 1 [file ol6c02534_si_001.pdf]

Supporting Information

An Atypical Flavin-Containing Monooxygenase Homologue Catalyzes the Third Epoxidation in the Verrucosidin Biosynthesis

Hui-Ling Wei,[†a] Xiao-Ling Chen,[†a] Wen-Bing Yin,[b] Jie Fan,[c] and Shu-Ming Li*[a]

Philipps-Universität Marburg, Fachbereich Pharmazie, Institut für Pharmazeutische Biologie und Biotechnologie, Robert-Koch-Straße 4, 35037 Marburg, Germany

[a] Philipps-Universität Marburg, Fachbereich Pharmazie, Institut für Pharmazeutische Biologie und Biotechnologie, Robert-Koch-Straße 4, 35037 Marburg, Germany

[b] State Key Laboratory of Mycology, Institute of Microbiology Chinese Academy of Sciences, Beijing, 100101 P. R. China

[c] Department of Microbiology, College of Life Science, Nankai University, Tianjin, 300071 P. R. China

[†]These authors contributed equally

*Corresponding author. Email: shuming.li@staff.uni-marburg.de

Table of Contents

Experiment Procedures 1

1. Strains, media, and culture conditions 1

2. Genomic DNA isolation and PCR 1

3. Plasmid construction 1

4. Genetic manipulation of *P. polonicum* 1

5. Heterologous expression in *A. nidulans* 1

6. Fermentation, extraction, and isolation of metabolites 1

7. HPLC equipment for analysis and substance isolation 1

8. LC-MS analysis for secondary metabolites 2

9. NMR analysis 2

10. Structural elucidation 2

11. Feeding experiments 2

12. Phylogenetic tree construction 2

13. AlphaFold-based structural prediction 2

14. Physiochemical properties of the compounds described in this study 2

Supplementary Tables 3

Table S1. Strains used and constructed in this study 3

Table S2. Plasmids used and constructed in this study. 4

Table S3. Primers used in this study. 5

Table S4. Comparison of proteins encoded by the *vec* cluster in CGMCC 3.15272 and *dov* cluster in NRRL995 7

Table S5. NMR data of verrucosidin (**1**) and normethylverrucosidin (**2**) in $CDCl_3$. 8

Table S6. Selected VecG homologues and experimentally validated FMO-related reference proteins were included for comparative analysis. 9

Supplementary Figures 13

Figure S1. Comparative LC-MS analysis of extracts from *P. polonicum* NRRL 995 and *P. polonicum* CGMCC 3.15272. 13

Figure S2. ^1H NMR spectrum of verrucosidin (**1**) in $CDCl_3$ (500 MHz). 14

Figure S3. ^1H NMR spectrum of normetylverrucosidin (**2**) in $CDCl_3$ (500 MHz). 15

Figure S4. Mass and UV spectra of compounds **1** and **2**. 16

Figure S5. Split-marker strategy for target gene disruption and PCR verification for *P. polonicum* strain. 17

Figure S6. Predicted domain architecture and topology features of VecC, VecG and VecG-related proteins (NtnK, AtnK, and AtnA) based on InterPro analysis. 18

Figure S7. Phylogenetic analysis of VecG sequence-related FMOs available in the Swiss-Prot database. 19

Figure S8. Multiple-sequence alignments of VecG and closely related homologues. 20

Figure S9. AlphaFold-based structural models of VecC, VecG, and DovG. 21

References 22

Experiment Procedures

1. Strains, media, and culture conditions

The strains, plasmids, and primers used in this study are listed in Tables S1–S3. *Penicillium polonicum* CGMCC 3.15272 was maintained on potato dextrose agar (PDA, 24 g/L potato dextrose broth, 1.6% (w/v) agar) plates at 25 °C for sporulation and cultivated on rice medium in 250 mL flasks for secondary metabolite production. Transformants were selected on PDA supplemented with hygromycin B (300 μg/mL) or G418 (300 μg/mL). *Aspergillus nidulans* strains were grown on glucose minimal medium (1.0% (w/v) glucose, 50 mL/L salt solution, 1 mL/L trace element solution, and 1.6% (w/v) agar) at 37 °C for sporulation and on PD medium for feeding experiments with appropriate auxotrophic supplements when required. *Escherichia coli* DH5α was cultured in LB medium at 37 °C with ampicillin (100 μg/mL) for plasmid construction. *Saccharomyces cerevisiae* BJ5464-npgA was grown in YPD medium at 30 °C, and SC-Ura medium was used for selection.

2. Genomic DNA isolation and PCR

Genomic DNA from *P. polonicum* and *A. nidulans* was isolated from mycelia grown in liquid medium using a previously described phenol/chloroform-based protocol.[1] PCR amplification was performed using Phusion High-Fidelity DNA polymerase (NEB) with primers listed in Table S3.

3. Plasmid construction

Plasmids for gene deletion and heterologous expression were constructed by homologous recombination in *E. coli* or *S. cerevisiae* as described previously.[1] For gene deletion in *P. polonicum*, split-marker constructs were generated by using approximately 1.5 kb upstream and downstream flanking regions of each target gene fused to hygromycin or G418 resistance cassettes. For heterologous expression in *A. nidulans* LO8030, genomic fragments from *P. polonicum* were amplified and cloned into vector pJN017 under control of the *gpdA* promoter at the *wA* locus by homologous recombination.

4. Genetic manipulation of *P. polonicum*

Gene deletion mutants of *P. polonicum* were generated by PEG-mediated protoplast transformation following a modified published procedure.[1] Hygromycin or G418-resistant colonies were purified on selective PDA plates and verified by diagnostic PCR.

5. Heterologous expression in *A. nidulans*

PEG-mediated protoplast transformation of *A. nidulans* LO8030 was carried out with auxotrophic selection according to a previously reported method.[1] The corresponding expression plasmids were introduced individually to generate the recombinant strains used for feeding experiments.

6. Fermentation, extraction, and isolation of metabolites

For metabolite production, fungal strains were cultivated on rice medium at 25 °C for 14 days.[1] Cultures were extracted twice with ethyl acetate, and the organic phases were concentrated under reduced pressure to yield crude extracts. Target compounds were isolated by preparative or semi-preparative HPLC using mixtures of acetonitrile and water as the mobile phases.

7. HPLC equipment for analysis and substance isolation

Preparative and semi-preparative HPLC separations were carried out according to previously reported methods,[1] using the same instrumentation and columns.

8. LC-MS analysis for secondary metabolites

LC-MS data were acquired using the same instrument and analytical method as previously reported,[1] with a 30 min gradient applied for all analyses.

9. NMR analysis

The isolated compounds were dissolved in $CDCl_3$, and their NMR spectra were acquired using the previously reported spectrometer (JEOL ECA-500 MHz spectrometer) and software (MestReNov.14.2.1).[1] Chemical shifts were referenced to the residual solvent signals.

10. Structural elucidation

The structures of the known compounds **1** and **2** were determined by comparing their MS and NMR data with those reported in the literature.[2–4]

11. Feeding experiments

For feeding experiments, the precursors deoxyverrucosidin or nordeoxyverrucosidin were dissolved in DMSO to give 50 mM stock solutions. The fungal strains were first cultivated in 5 mL PD media for 2 days. 10 µL stock solutions were subsequently added to the cultures, yielding a final concentration of about 0.1 mM. After cultivation at room temperature for a further 5 days, the secondary metabolites were extracted with EtOAc twice and dissolved in methanol for LC-MS analysis.

12. Phylogenetic tree construction

Protein sequences used for phylogenetic analysis were obtained from Swiss-Prot by BLAST searches using the protein VceG as the query. The sequence alignments and phylogenetic analysis were performed with MEGA (version 12) using the maximum likelihood method. The resulting tree was visualized and edited for presentation.

13. AlphaFold-based structural prediction

The amino acid sequences of VecC, VecG, and DovG were submitted individually to the AlphaFold Server (https://alphafoldserver.com) for structure prediction using default settings. The predicted structures were exported from the AlphaFold Server and used to compare the overall domain organization of these enzymes.

14. Physiochemical properties of the compounds described in this study

verrucosidin (**1**): pale yellow oil, ^{1}H NMR data are given in Table S5 and ^{1}H NMR spectrum in Figure S2; HRMS(ESI) m/z: $[M + H]^+$ calcd. for $C_{24}H_{33}O_6$ 417.2277; found 417.2295.

normethylverrucosidin (**2**): yellow oil, ^{1}H NMR data are given in Table S5 and ^{1}H NMR spectrum in Figure S3, HRMS(ESI) m/z: $[M + H]^+$ calcd. for $C_{23}H_{31}O_6$ 403.2121; found 403.2128.

Supplementary Tables

Table S1. Strains used and constructed in this study.

Strain	Genotype	Source
E. coli		
DH5α	F⁻*endA1 glnV44 thi-1 recA1 relA1 gyrA96 deoR nupG purB20* φ80dl*acZ*ΔM15 Δ(*lacZYA-argF*)U169, hsdR17($r_K^- m_K^+$), λ⁻	[5]
BL21(DE3)	*fhuA2 [lon] ompT gal (λ DE3) [dcm] ΔhsdS* *λ DE3 = λ sBamHIo ΔEcoRI-B int::(lacI::PlacUV5::T7 gene1) i21 Δnin5*	[6]
S. cerevisiae		
BJ5464-npgA	*MATα ura3-52 leu2-Δ1 trp1 his3-Δ200 pep4::HIS3 prb1-Δ1.6R can1 GAL npgA*	[7]
Aspergillus nidulans		
LO8030	*pyroA4, riboB2, pyrG89, nkuA::argB*, deletion of 9 biosynthetic gene clusters	[8]
BK06	Δ*wA*-PKS::*gpdA*(p)-*Afribo* in LO8030	[9]
HLW79	Δ*wA*-PKS::*gpdA*(p):*vecG-Afribo* in LO8030	This study
HLW87	Δ*wA*-PKS::*gpdA*(p):*dovG-Afribo* in LO8030	This study
HLW104	Δ*wA*-PKS::*gpdA*(p):*vecG*(I301T)-*Afribo* in LO8030	This study
HLW105	Δ*wA*-PKS::*gpdA*(p):*vecG*(H572R)-*Afribo* in LO8030	This study
HLW109	Δ*wA*-PKS::*gpdA*(p):*vecG*(L337F)-*Afribo* in LO8030	This study
Penicillium polonicum		
NRRL 995	wild type	NRRL*
CGMCC 3.15272	wild type	CGMCC*
HLW120	Δ*vecC*	This study
HLW121	Δ*vecD*	This study
HLW122	Δ*vecE*	This study
HLW123	Δ*vecG*	This study
HLW124	Δ*vecC*Δ*vecG*	This study
HLW125	Δ*vecG*-P1 (Δ*vecG*-FMO)	This study
HLW126	Δ*vecG*-P2 (Δ*vecG*-membrane)	This study
HLW127	Δ*vecC*Δ*vecG*-P2 (Δ*vecC*Δ*vecG*-membrane)	This study

*NRRL: ARS Culture Collection

*CGMCC: China General Microbiological Culture Collection

Table S2. Plasmids used and constructed in this study.

Plasmid	Description	Source
pJN017	*URA3, wA flanking, gpdA*(*p*), *afriboB, ampR*	[9]
p5HY	Two-third of the *hph* resistance gene at the 5´-end, originated from the pUChph and inserted into pESC-URA. For gene replacement using *hph* as selection marker	[10]
p3YG	Two-third of the hph resistance gene at the 3´-end, originated from the pUChph and inserted into pESC-URA. For gene replacement using *hph* as selection marker.	[10]
pAG1-G418	*hph* (hygromycin marker), *neo* (G418 marker), *Kana*	[11]
pHLW10 (p5HY-*verC1*)	a 1458 bp upstream PCR fragment of *dovG* from genomic DNA of *P. polonicum* NRRL 995 inserted in p5HY	[1]
pHLW11 (p3YG-*verC1*)	a 1225 bp downstream PCR fragment of *dovG* from genomic DNA of *P. polonicum* NRRL 995 inserted in p3YG	[1]
pHLW21 (p5HY-*verC2*)	a 1260 bp upstream PCR fragment of *dovC* from genomic DNA of *P. polonicum* NRRL 995 inserted in p5HY	[1]
pHLW22 (p3YG-*verC2*)	a 1187 bp downstream PCR fragment of *dovC* from genomic DNA of *P. polonicum* NRRL 995 inserted in p3YG	[1]
pHLW79 (pJN017-*vecG*-CGMCC)	a 3463 bp fragment of *vecG* with its terminator from genomic DNA of *P. polonicum* NRRL CGMCC 3.15272 inserted in pJN017	This study
pHLW83 (pG418-*verC2*)	a 1445 bp upstream PCR fragment of *vecC* from genomic DNA of *P. polonicum* CGMCC 3.15272 inserted in pAG1-G418	This study
pHLW84 (pG418-*verC2*)	a 1442 bp downstream PCR fragment of *vecC* from genomic DNA of *P. polonicum* CGMCC 3.15272 inserted in pAG1-G418	This study
pHLW87 (pJN017-*dovG*-NRRL995)	a 3463 bp fragment of *dovG* with its terminator from genomic DNA of *P. polonicum* NRRL 995 inserted in pJN017	This study
pHLW98 (p5HY-*verC1*-membrane)	a 1149 bp upstream PCR fragment of the transmembrane region of *vecG* from genomic DNA of *P. polonicum* CGMCC 3.15272 inserted in p5HY	This study
pHLW99 (p3YG-*verC1*-FMO)	a 1176 bp downstream PCR fragment of the FMO core of *vecG* from genomic DNA of *P. polonicum* CGMCC 3.15272 inserted in p3YG	This study
pHLW104 (pJN017-*vecG*-I301T-CGMCC)	a 3463 bp fragment of *vecG* carrying the I301T mutation, together with its terminator from genomic DNA of *P. polonicum* CGMCC 3.15272 inserted in pJN017	This study
pHLW105 (pJN017-*vecG*-H572R-CGMCC)	a 3463 bp fragment of *vecG* carrying the H572R mutation, together with its terminator from genomic DNA of *P. polonicum* CGMCC 3.15272 inserted in pJN017	This study
pHLW109 (pJN017-*vecG*-L337F-CGMCC)	a 3463 bp fragment of *vecG* carrying the L337F mutation, together with its terminator from genomic DNA of *P. polonicum* CGMCC 3.15272 inserted in pJN017	This study

Table S3. Primers used in this study.

Primer	Oligonucleotide sequence 5'-3'	Function
HLW_pHLW10_F	AAGAATTGTTAATTAAGAGCTCAGATCcacgcgaagtgttcagtatgag	Amplification of the upstream region of *vecG* and 2/3 from the *hph* gene (5'end) from pHLW10 for split marker
HLW_5-Hygsplit-R	TCGAAGTAGCGCGTCTGC	
HLW_3-Hygsplit-F	TTGGGGAATTCAGCGAGAGC	Amplification of the downstream region of *vecG* and 2/3 from the *hph* gene (3'end) from pHLW11 for split marker
HLW_pHLW11_R	TAGCCGCGGTACCAAGCTTACTCGAgcgtagttagttgcactagtcg	
HLW_verC1-V-F	ctggccttactctagccagtg	Screening of Δ*vecG* transformants
HLW_verC1-V-R	gcgaggttctcttccgtaacag	Screening of Δ*vecG* transformants
HLW_verC1-V-F2	cgagcctagatgctgtcattg	Screening of Δ*vecG* transformants
HLW_verC1-V-R2	ctctgccacttcttggctattc	Screening of Δ*vecG* transformants
HLW_verC2-p5HY-F	AAGAATTGTTAATTAAGAGCTCAGATCcgttcgattcactctcgcttatg	Amplification of the upstream region of *vecC* and 2/3 from the *hph* gene (5'end) from pHLW21 for split marker
HLW_verC2-p3YG-R	TAGCCGCGGTACCAAGCTTACTCGAgaatcatcagaatgtccacagcag	Amplification of the downstream region of *vecC* and 2/3 from the *hph* gene (3'end) from pHLW22 for split marker
HLW_verC2-F	caattctcacacggcaacaac	Screening of Δ*vecC* transformants
HLW_verC2-R	ggtcgtcttccaaggcatatc	Screening of Δ*vecC* transformants
HLW_verB-inside-R	catggtctatggatggcactg	Screening of Δ*vecC* transformants
HLW_verC2-V-R	gcatctggtctgtcattgatgg	Screening of Δ*vecC* transformants
HLW_pHLW79-F	AACAGCTACCCCGCTTGAGCAGACATCACCatgactttccgagtgatcattg ttg	Amplification of the *vecG* gene and its terminator from *P. polonicum* CGMCC 3.15272 to construct pHLW79; Amplification of the *dovG* gene and its terminator from *P. polonicum* NRRL 995 to construct pHLW87
HLW_pHLW79-R	TCAACACCATATTTTAATCCCATGTGGGCGCCcgtctctctggtacgactc atg	
HLW_pHLW83-F	TCGCGGCCGGCCGGCGCGCCGTTTAAACGGATTTgctccagcaacc gtatcaac	Amplification of the upstream region of *vecC* from *P. polonicum* CGMCC 3.15272 to construct pHLW83
HLW_pHLW83-R	ATTGTAAGCGTTAATCTAGAATTAATTAATTTcacaaggacgttgctggtat c	
HLW_pHLW84-F	TCCTATTCCGAAGTTCCTATTCTCTAGAgtgttgctgtgaagagaatgcg	Amplification of the downstream region of *vecC* from *P. polonicum* CGMCC 3.15272 to construct pHLW83
HLW_pHLW84-R	AGAATTAAGGGAGTCACGAAGCTTCGACgcatctggtctgtcattgatgg	

Table S3. Primers used in this study (continued).

HLW_G418-Split-F	CTCGACGTTGTCACTGAAGC	Amplification of 2/3 from the *G418* gene (5'end) for split marker cloning
HLW_G418-Split-R	CTTCCATCCGAGTACGTGCTC	Amplification of 2/3 from the *G418* gene (3'end) for split marker cloning
HLW_pHLW98-F	AAGAATTGTTAATTAAGAGCTCAGATCggactcatggatccggacaag	Amplification of the upstream region of the transmembrane region of *vecG* from *P. polonicum* CGMCC 3.15272 to construct pHLW98
HLW_pHLW98-R	ACCCTCACTAAAGGGCGGCCgcgactcatagttcgtctcgtag	
HLW_pHLW99-F	ACTCACTATAGGGCCCGGGCGctgccttgcgaggttatatctg	Amplification of the downstream region of the FMO core of *vecG* from *P. polonicum* CGMCC 3.15272 to construct pHLW99
HLW_pHLW99-R	TAGCCGCGGTACCAAGCTTACTCGActaggcagtacacaccaagc	
HLW_verC1-FMO-F	ggcgtttaccgaatgtatgatg	Screening of Δ*vecG*-P1 transformants
HLW_verC1-FMO-R	ggcatccacaatagctgtgg	Screening of Δ*vecG*-P1 transformants
HLW_verC1-FMO-V-R	ctgcgaatcggattgatcaac	Screening of Δ*vecG*-P1 transformants
HLW_verC1-M-F	ccaggcagtctcatcttcaattg	Screening of Δ*vecG*-P2 transformants
HLW_verC1-M-R	gcctcttatgagcgaggatattc	Screening of Δ*vecG*-P2 transformants
HLW_verC1-M-V-F	gagtagcagttcagtgtgctg	Screening of Δ*vecG*-P2 transformants
HLW_pHLW104-F	agtggtcgtggggtcgtaccgtctgcgtcggcgatagcattc	I301T mutation of the *vecG* gene from *P. polonicum* CGMCC 3.15272
HLW_pHLW104-R	tgctatcgccgacgcagacggtacgaccccacgaccactg	
HLW_pHLW105-F	cGcgtcagccctttgtattac	H572R mutation of the *vecG* gene from *P. polonicum* CGMCC 3.15272
HLW_pHLW105-R	gtaatacaaagggctgacgCg	
HLW_pHLW109-F	gcgatgttTgaccacagcccag	L337F mutation of the *vecG* gene from *P. polonicum* CGMCC 3.15272
HLW_pHLW109-R	ctgggctgtggtcAaacatcgc	

Table S4. Comparison of proteins encoded by the *vec* cluster in CGMCC 3.15272 and *dov* cluster in NRRL995

P. polonicum CGMCC 3.15272	Length	Location at JAPDKX010000002.1	Identity	Putative function
VecA	2566 aa	(30001-36548, 36605-37757)	99.8% to DovA	Polyketide synthase
VecB	228 aa	(22311-22997)	99.6% to DovB	Methyltransferase
VecC	480 aa	(23759-24055, 24104-24410, 2470-24838, 24900-25366)	95.2% to DovC	Flavin-containing monooxygenase
VecD	368 aa	(25525-26272, 26337-26692)	99.5% to DovD	Epoxide expandase
VecE	520 aa	(27469-27489, 27544-27571, 27633-27696, 27758-29207)	99.1% to DovE	Cytochrome P450
VecF	576 aa	(39476-39870, 39928-40223, 40278-40384, 40436-40689, 40739-41417)	97.4% to DovF	Transcription Factor
VecG	805 aa	(18892-19639, 19709-19867, 19927-20878, 20933-21201, 21261-21346, 21403-21447, 21502-21594, 21648-21713)	99.6% to DovG	Flavin-containing monooxygenase

*CGMCC: China General Microbiological Culture Collection Center

Table S5. NMR data of verrucosidin (**1**) and normethylverrucosidin (**2**) in $CDCl_3$.

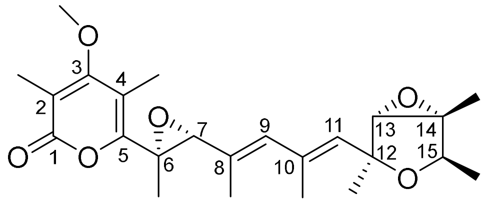

verrucosidin (**1**)

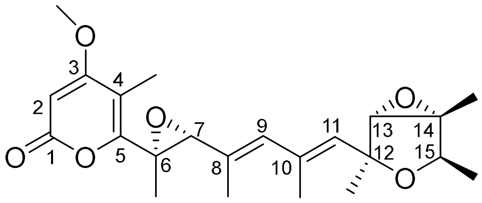

normethylverrucosidin (**2**)

Position	δ_H, multi., *J* in Hz	δ_H, multi., *J* in Hz
2-H or CH_3	2.02, s	5.50, s
7	3.46, br s	3.50, br s
9	5.83, br s	5.86, br s
11	5.44, br s	5.47, br s
13	3.41, s	3.44, s
15	4.10, q, 6.8	4.12, q, 6.8
3-OCH_3	3.80, s	3.84, s
4-CH_3	2.01, s	2.01, s
6-CH_3	1.40, s	1.43, s
8-CH_3	1.88, br s	1.91, br s
10-CH_3	1.93, br s	1.96, br s
12-CH_3	1.39, s	1.42, s
14-CH_3	1.45, s	1.48, s
15-CH_3	1.16, d, 6.8	1.20, d, 6.8

The ^{1}H NMR data of **1** and **2** correspond well to those of verrucosidin and normethylverrucosidin, respectively.[2–4]

Table S6. Selected VecG homologues and experimentally validated FMO-related reference proteins were included for comparative analysis.

Accession	Species	Length (aa)	Identity to VecG (%)	Query Coverage (%)	Annotation
VecG	*P. polonicum* CGMCC 3.15272	805	100	100	FAD-dependent monooxygenase (This study)
DovG	*P. polonicum* NRRL995	805	99.6	100	FAD-dependent monooxygenase (inactive *in vivo*)
VerC1: A0A1V6NWP3.1	*P. polonicum* X6	805	99.75	100	FAD-dependent monooxygenase (uncharacterized)
NtnK: A0A455M7R7.1	*Nectria sp.*	760	30.93	98	FAD-dependent monooxygenase
AtnK: A0A455LLW7.1	*Arthrinium sp.*	756	30.5	98	FAD-dependent monooxygenase
AtnA: A0A455LLW3.1	*Arthrinium sp.*	751	30.75	54	FAD-dependent monooxygenase
CAI7659374.1	*Penicillium glandicola*	809	85.04	100	unnamed protein product
KAH6989817.1	*Ilyonectria sp.* MPI-CAGE-AT-0026	809	48.01	100	hypothetical protein
KAH7011009.1	*Ilyonectria destructans*	809	47.76	100	hypothetical protein
KAH7147162.1	*Dactylonectria estremocensis*	818	47.97	100	hypothetical protein
KAH6989863.1	*Ilyonectria destructans*	809	47.89	100	hypothetical protein
KAH7231139.1	*Fusarium tricinctum*	823	48.49	99	hypothetical protein
XP_046041176.1	*Fusarium redolens*	823	48.62	99	uncharacterized protein
KAH9203318.1	*Leptodontidium sp.* 2 PMI_412	800	49.87	96	hypothetical protein
EXK27523.1	*Fusarium oxysporum f. sp.* melonis 26406	845	48.24	99	hypothetical protein
KAH7215360.1	*Fusarium oxysporum*	823	48.12	99	hypothetical protein
KAL7755754.1	*Fusarium oxysporum f. sp.* zingiberi	835	47.9	99	hypothetical protein
KAH7176063.1	*Dactylonectria macrodidyma*	811	48.94	99	hypothetical protein
CAG7556528.1	*Fusarium equiseti*	792	47.74	99	unnamed protein product
KAF4415470.1	*Fusarium acutatum*	842	48.12	99	FAD NAD(P)-binding domain-containing
XP_046093436.1	*Ilyonectria robusta*	795	46.77	100	uncharacterized protein
KAF2664645.1	*Microthyrium microscopicum*	819	45.85	99	FAD/NAD(P)-binding domain-containing protein
KAF4334071.1	*Fusarium beomiforme*	823	46.49	100	FAD NAD(P)-binding domain protein
KAK3311782.1	*Apodospora peruviana*	826	45.26	99	hypothetical protein
KAK3317898.1	*Apodospora peruviana*	855	45.26	99	hypothetical protein
KAH6876830.1	*Thelonectria olida*	774	46.23	99	hypothetical protein

Table S6. Selected VecG homologues and experimentally validated FMO-related reference proteins included for comparative analysis (continued).

Accession	Species	Length (aa)	Identity to VecG (%)	Query Coverage (%)	Annotation
KAG4275017.1	*Fusarium proliferatum*	772	44.97	99	FAD binding domain-containing protein
KAF2418752.1	*Tothia fuscella*	823	43.25	99	FAD/NAD(P)-binding domain-containing protein
KAK0704287.1	*Lasiosphaeris hirsuta*	825	44.96	99	hypothetical protein
KAK3942865.1	*Diplogelasinospora grovesii*	818	43.55	100	hypothetical protein
KAL2132247.1	*Chaetomium olivicolor*	822	41.31	100	hypothetical protein
KAN3061841.1	*Chaetomium globosum*	822	42.57	99	FAD-dependent monooxygenase verC1
KAK4466004.1	*Cladorrhinum samala*	838	44.75	99	hypothetical protein
KAK4445725.1	*Podospora aff.* communis PSN243	841	42.5	99	hypothetical protein
KAK1750051.1	*Echria macrotheca*	819	40.8	99	hypothetical protein
KAJ9417961.1	*Fusarium oxysporum*	786	43.59	99	hypothetical protein
KAK0652570.1	*Cercophora newfieldiana*	845	42.8	99	hypothetical protein
RDI87660.1	*Venturia inaequalis*	816	41.23	99	hypothetical protein
KAK3349693.1	*Lasiosphaeria hispida*	834	43.98	99	FAD binding domain protein
KAE9978828.1	*Venturia inaequalis*	816	41.12	99	hypothetical protein
KAE9965136.1	*Venturia inaequalis*	816	41.12	99	hypothetical protein
XP_070921440.1	*Madurella fahalii*	856	41.75	100	FAD NAD(P)-binding domain protein
KAH6855970.1	*Chaetomium sp.* MPI-CAGE-AT-0009	1008	42.77	99	hypothetical protein
KAK3363197.1	*Lasiosphaeria hispida*	846	42.39	100	FAD binding domain protein
KAL2015409.1	*Thermocarpiscus australiensis*	829	44.09	100	hypothetical protein
KAK3348913.1	*Lasiosphaeria hispida*	786	44.13	97	hypothetical protein
KAL2143755.1	*Corynascus sepedonium*	824	41.3	100	hypothetical protein
XP_062637845.1	*Dichotomopilus funicola*	822	41.49	100	uncharacterized protein
KAH0280443.1	*Aureobasidium melanogenum*	1312	40.75	99	FAD/NAD(P)-binding domain-containing protein, partial
KAK4247280.1	*Corynascus novoguineensis*	824	41.42	100	hypothetical protein
KAH6634761.1	*Chaetomium sp.* MPI-SDFR-AT-0129	822	41.13	100	hypothetical protein
XP_007781688.1	*Coniosporium apollinis* CBS 100218	828	41.48	99	uncharacterized protein
KAG9959676.1	*Aureobasidium melanogenum*	1312	40.5	99	FAD/NAD(P)-binding domain-containing protein, partial

Table S6. Selected VecG homologues and experimentally validated FMO-related reference proteins included for comparative analysis (continued).

Accession	Species	Length (aa)	Identity to VecG (%)	Query Coverage (%)	Annotation
KAG9604685.1	*Aureobasidium melanogenum*	811	40.42	99	FAD/NAD(P)-binding domain-containing protein, partial
KAG7287254.1	*Staphylotrichum longicolle*	1084	41.32	98	hypothetical protein
KAL1842350.1	*Mycothermus thermophilus*	827	40.59	99	hypothetical protein
THW37037.1	*Aureobasidium pullulans*	872	40.35	98	FAD/NAD(P)-binding domain-containing protein
KAK4041625.1	*Parachaetomium inaequale*	1529	42.47	100	Neuroligin-4, X-linked
THZ96045.1	*Aureobasidium pullulans*	807	40.25	98	FAD/NAD(P)-binding domain-containing protein
XP_064667400.1	*Canariomyces notabilis*	835	41.34	98	FAD/NAD(P)-binding domain-containing protein
XP_070866076.1	*Remersonia thermophila*	834	40.49	100	hypothetical protein
THW57178.1	*Aureobasidium pullulans*	807	40.25	98	FAD/NAD(P)-binding domain-containing protein
XP_040875696.1	*Aureobasidium melanogenum* CBS 110374	805	40.49	99	FAD/NAD(P)-binding domain-containing protein
GAB7353290.1	*Dothideomycetes sp.* NU459	795	40.64	99	hypothetical protein
KAK3896965.1	*Staphylotrichum tortipilum*	824	42.1	100	hypothetical protein
KAH0339592.1	*Aureobasidium melanogenum*	837	40.42	100	FAD/NAD(P)-binding domain-containing protein, partial
XP_069220304.1	*Mycothermus thermophilus*	833	40.19	100	uncharacterized protein
KXX79717.1	*Madurella mycetomatis*	1515	41.57	99	Neuroligin-4, X-linked
KAH0336412.1	*Aureobasidium melanogenum*	784	40.12	100	FAD/NAD(P)-binding domain-containing protein
KAK4198200.1	*Triangularia verruculosa*	1497	40.12	100	Neuroligin-4, X-linked
KAK0618981.1	*Immersiella caudata*	784	41.58	94	hypothetical protein
MCJ1346261.1	*Peltigera leucophlebia*	823	40.59	99	hypothetical protein
KAL2153720.1	*Thermothelomyces myriococcoides*	823	41.85	100	hypothetical protein
KAK4239014.1	*Achaetomium macrosporum*	823	41.21	100	hypothetical protein
GAB6373432.1	*Madurella pseudomycetomatis*	851	40.27	98	hypothetical protein
XP_069201121.1	*Neodothiora populina*	842	40.37	100	uncharacterized protein
XP_046092944.1	*Ilyonectria robusta*	683	40.17	100	uncharacterized protein
KAL2170283.1	*Thermothelomyces hinnuleus*	823	41.11	100	hypothetical protein
KAL2165007.1	*Thermothelomyces fergusii*	824	42.2	99	hypothetical protein
XP_003652611.1	*Thermothielavioides terrestris* NRRL 8126	805	40.67	93	uncharacterized protein

Legend to Table S6: BLASTP analysis using VecG as the query against the NCBI non-redundant protein database, including all non-redundant GenBank CDS translations, identified 76 hits with query coverage >90% and sequence identity >40%. All 76 BLASTP-selected hits corresponded to proteins without experimental functional validation. Three additional FMO-related proteins (NtnK, AtnK, AtnA) with lower sequence identity to VecG were included as epoxidation-associated reference proteins,[12] although their roles have not been directly confirmed by biochemical assays.

Supplementary Figures

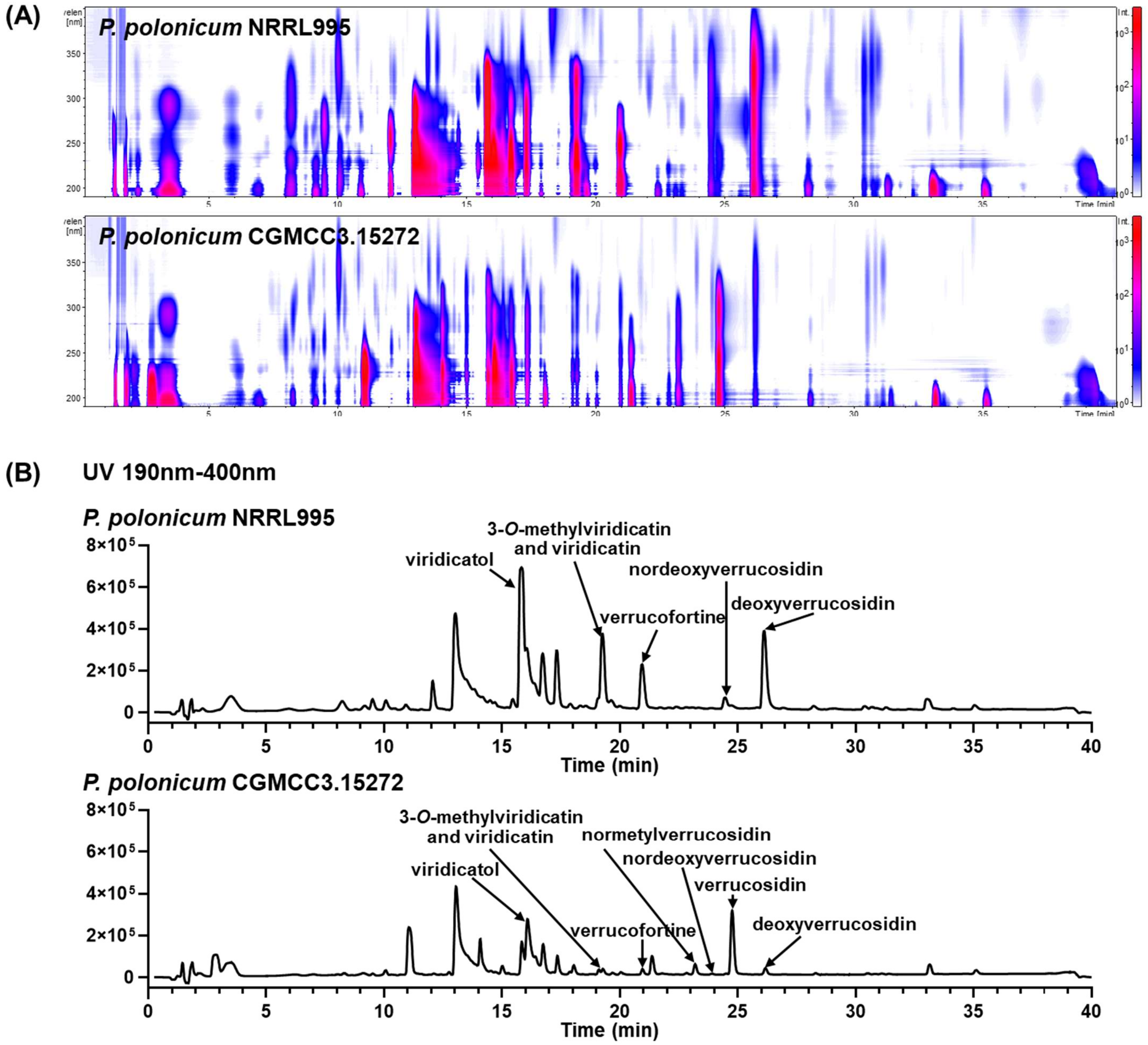

Figure S1. Comparative LC-MS analysis of extracts from *P. polonicum* NRRL 995 and *P. polonicum* CGMCC 3.15272.

(A) UV absorption spectra and (B) HPLC profiles of metabolites from *P. polonicum* NRRL 995 and *P. polonicum* CGMCC 3.15272 cultivated on rice medium for 7 days. UV absorption spectra were recorded over the range of 190–400 nm.

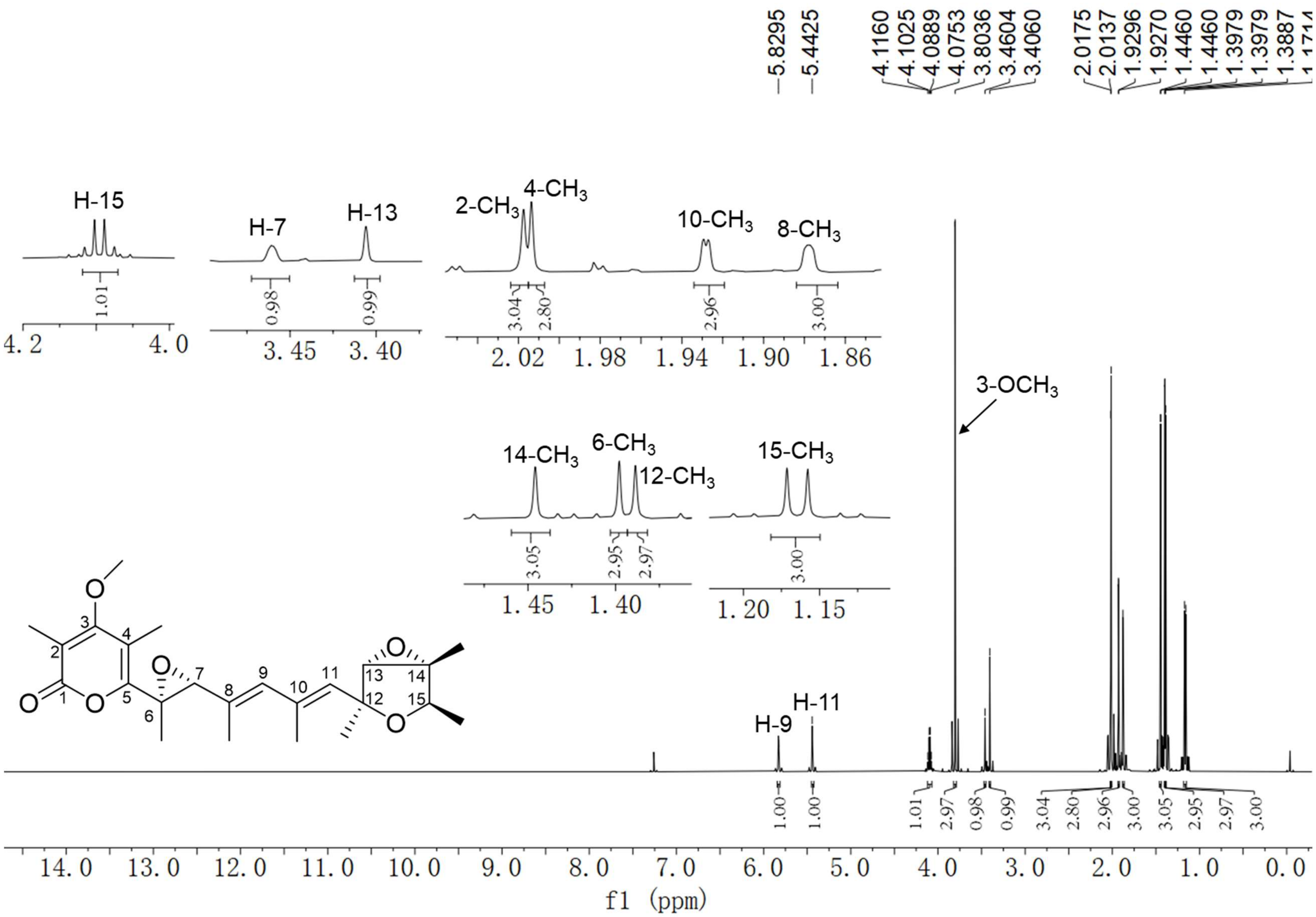

Figure S2. ^{1}H NMR spectrum of verrucosidin (**1**) in $CDCl_3$ (500 MHz).

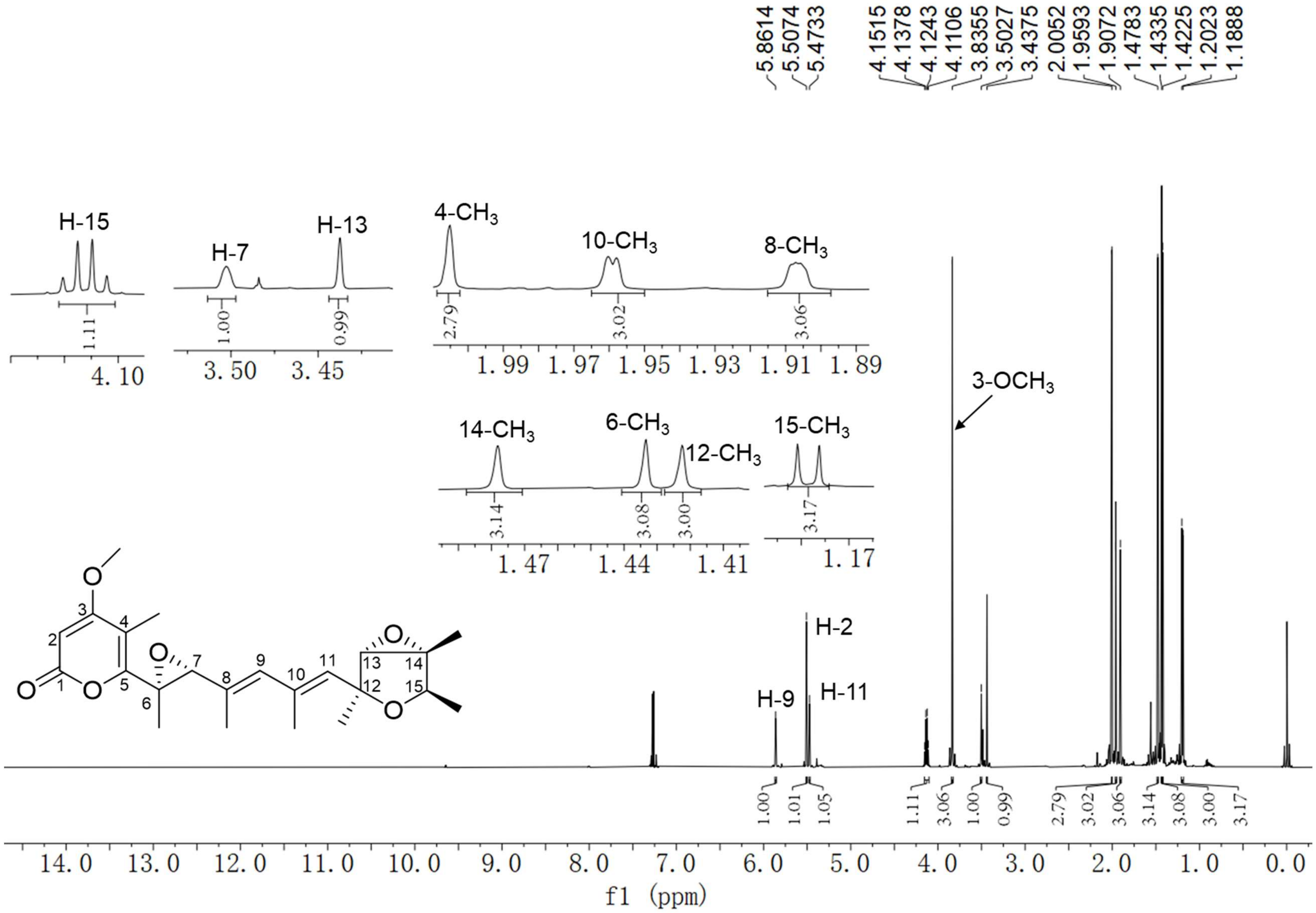

Figure S3. ^{1}H NMR spectrum of normetylverrucosidin (**2**) in $CDCl_3$ (500 MHz).

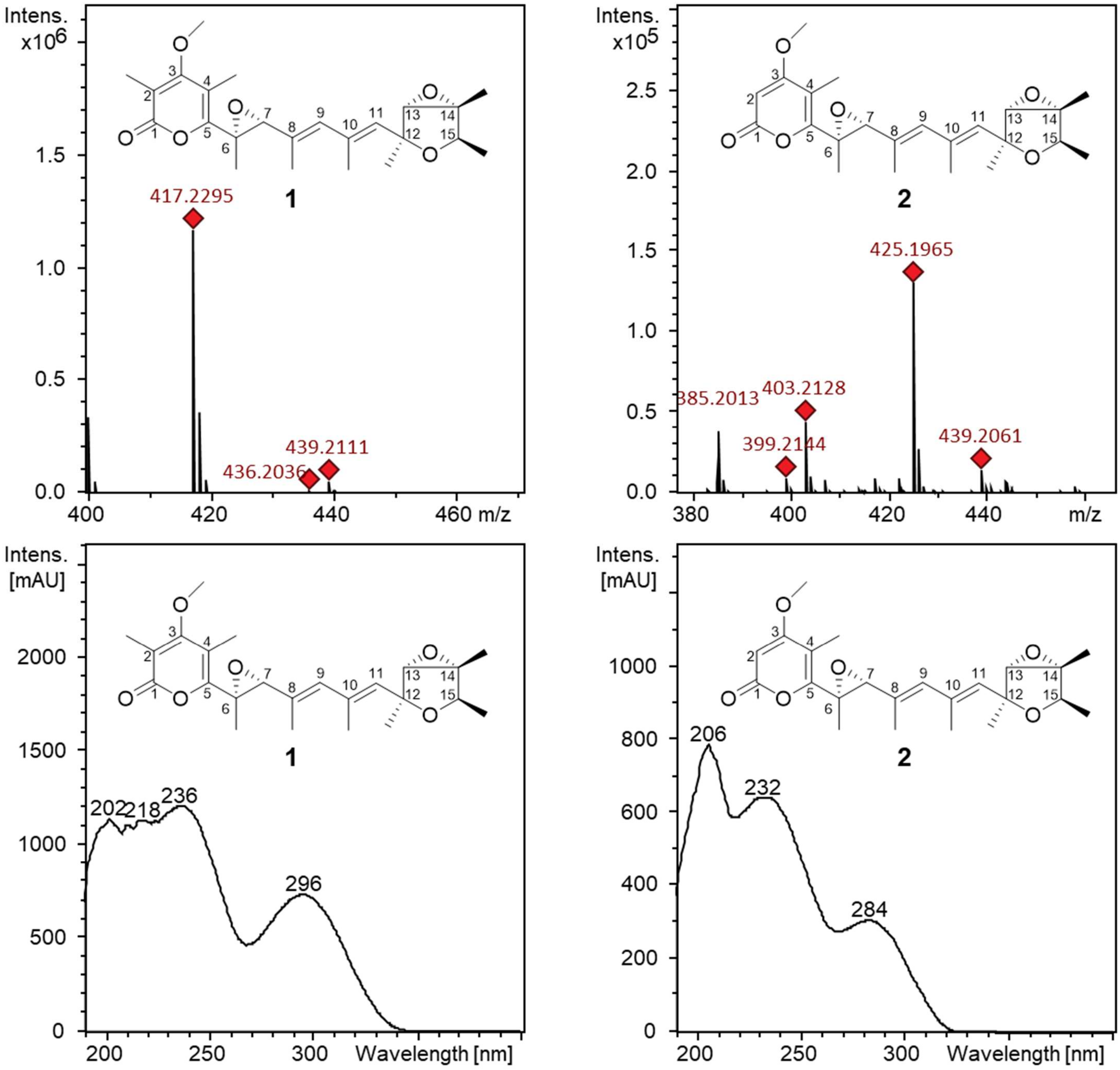

Figure S4. Mass and UV spectra of compounds **1** and **2**.

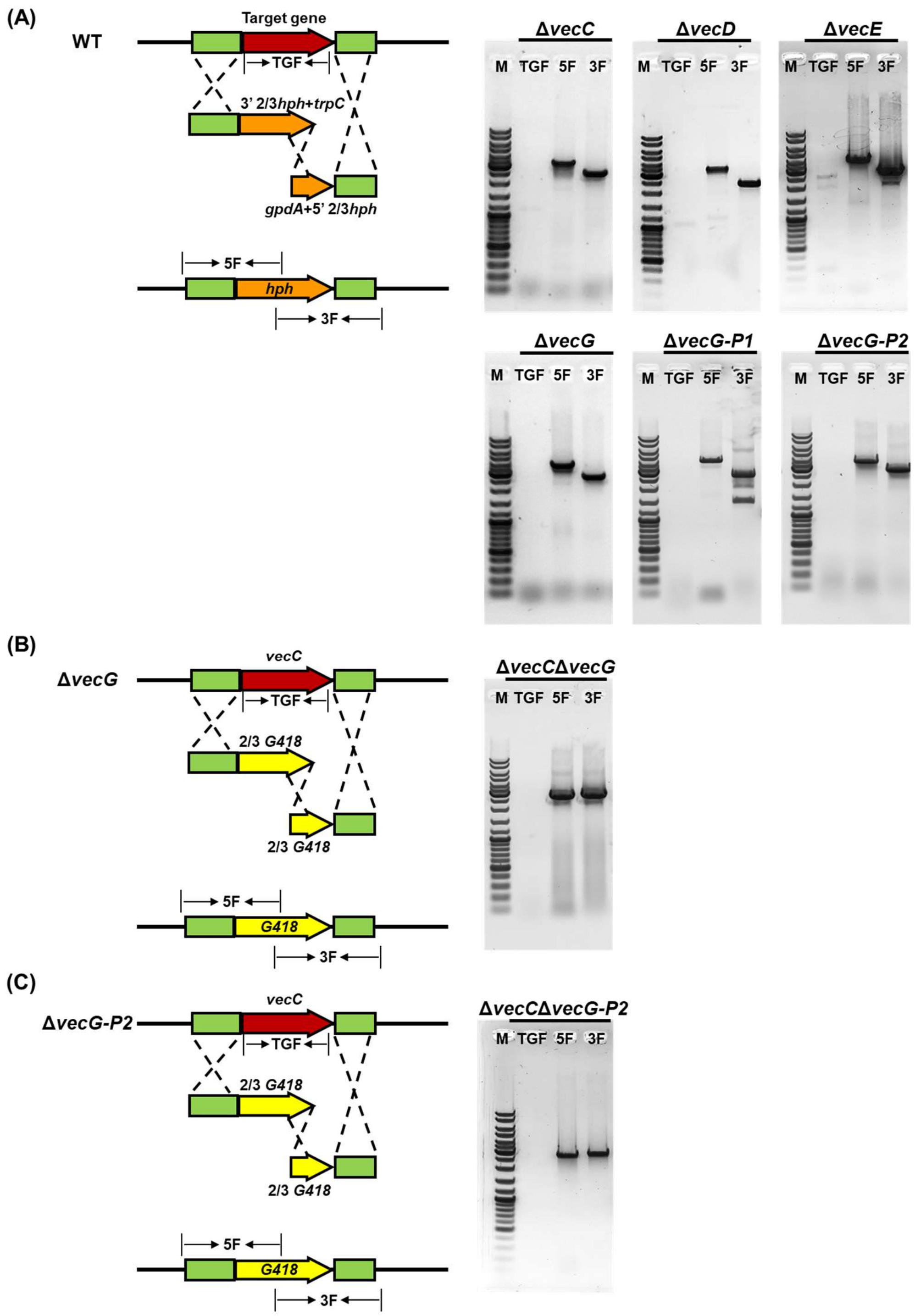

Figure S5. Split-marker strategy for target gene disruption and PCR verification for *P. polonicum* strain. (A) Target gene deletion in wild-type strain. (B) Deletion of *vecC* in Δ*vecG* mutant. (B) Deletion of *vecC* in Δ*vecG*-P2 mutant.

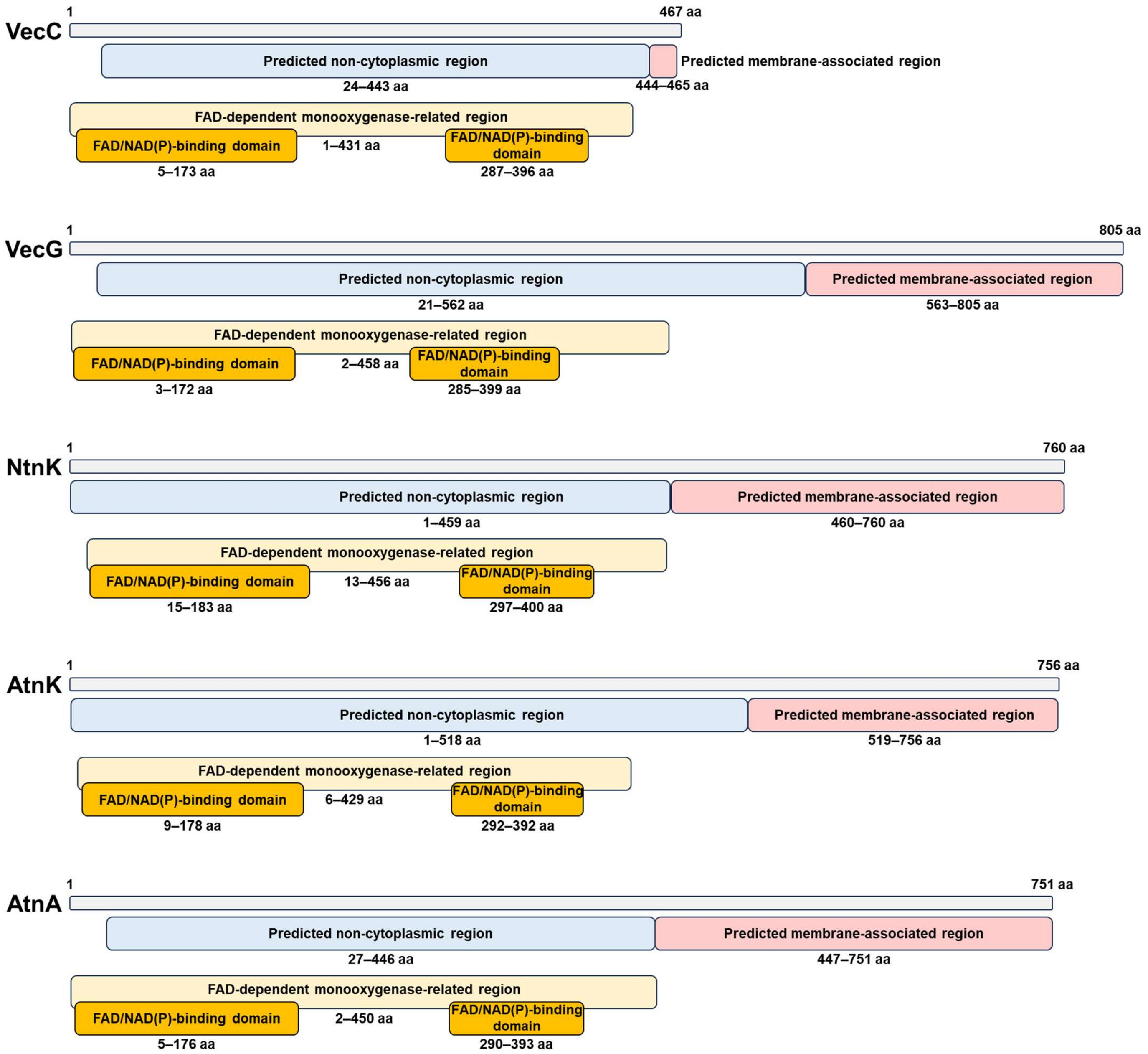

Figure S6. Predicted domain architecture and topology features of VecC, VecG and VecG-related proteins (NtnK, AtnK, and AtnA) based on InterPro analysis.

The full-length protein is shown schematically according to predicted amino acid positions. For VecC, InterPro analysis identified an FAD-dependent monooxygenase-related region spanning residues 1–431 with two FAD/NAD(P)-binding domains at aa 5–173 and 287–396. For VecG, InterPro analysis identified an FAD-dependent monooxygenase-related region spanning residues 2–458 with two FAD/NAD(P)-binding domains at aa 3–172 and 285–399. Topology-related predictions further indicated a broad non-cytoplasmic region from residues 21–562 and a C-terminal membrane-associated region from residues 563–805. All annotations represent bioinformatic predictions. NtnK, AtnK, and AtnA share a VecG-like architecture, comprising an FMO-like region and an N-terminal transmembrane region

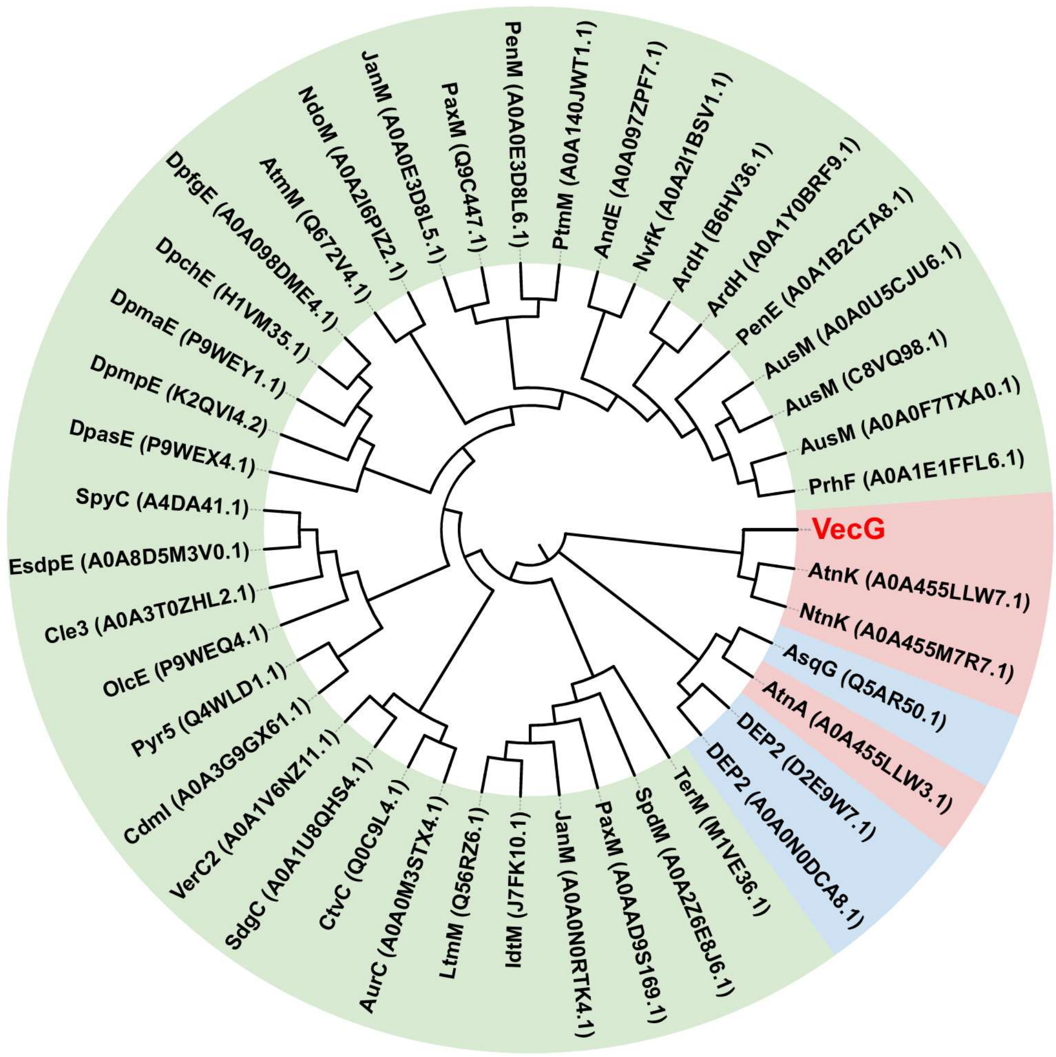

Figure S7. Phylogenetic analysis of VecG sequence-related FMOs available in the Swiss-Prot database. Protein names are followed by accession numbers in parentheses. Shaded regions indicate protein size groups: green, <500 aa; blue, 500–700 aa; pink, >700 aa. VecG is highlighted in red. These proteins have been reported to catalyze epoxidation reactions.

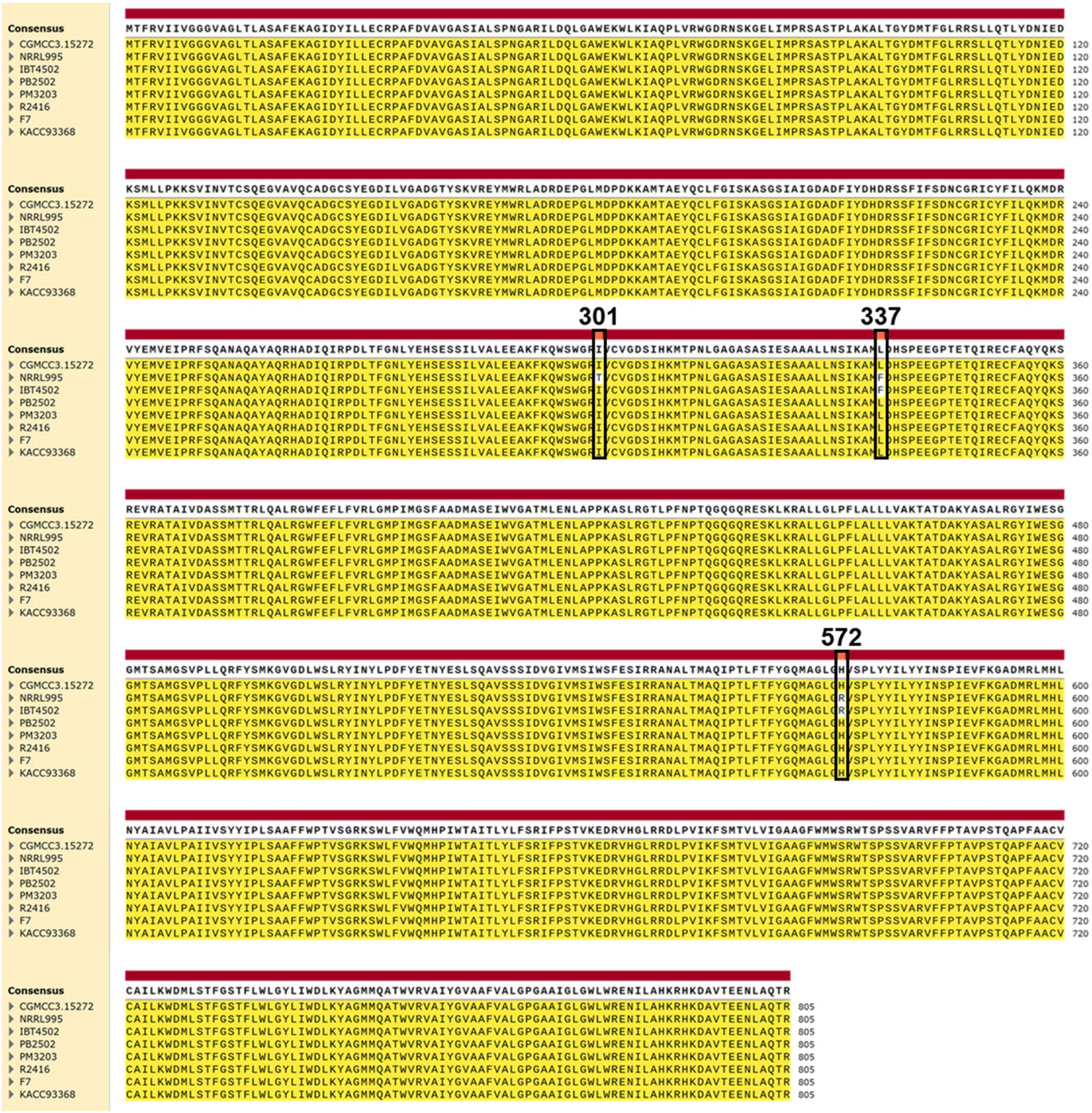

Figure S8. Multiple-sequence alignments of VecG and closely related homologues.

Protein sequences from *P. polonicum* CGMCC 3.15272, NRRL 995, IBT 4502, PB2502, PM3203, R2416, F7, and KACC 93368 were aligned to compare sequence conservation among VecG and related homologues. Conserved residues are highlighted in yellow, and black boxes indicate amino-acid positions showing sequence variation among the aligned homologues. The alignment shows high overall conservation, with only limited substitutions among the homologues.

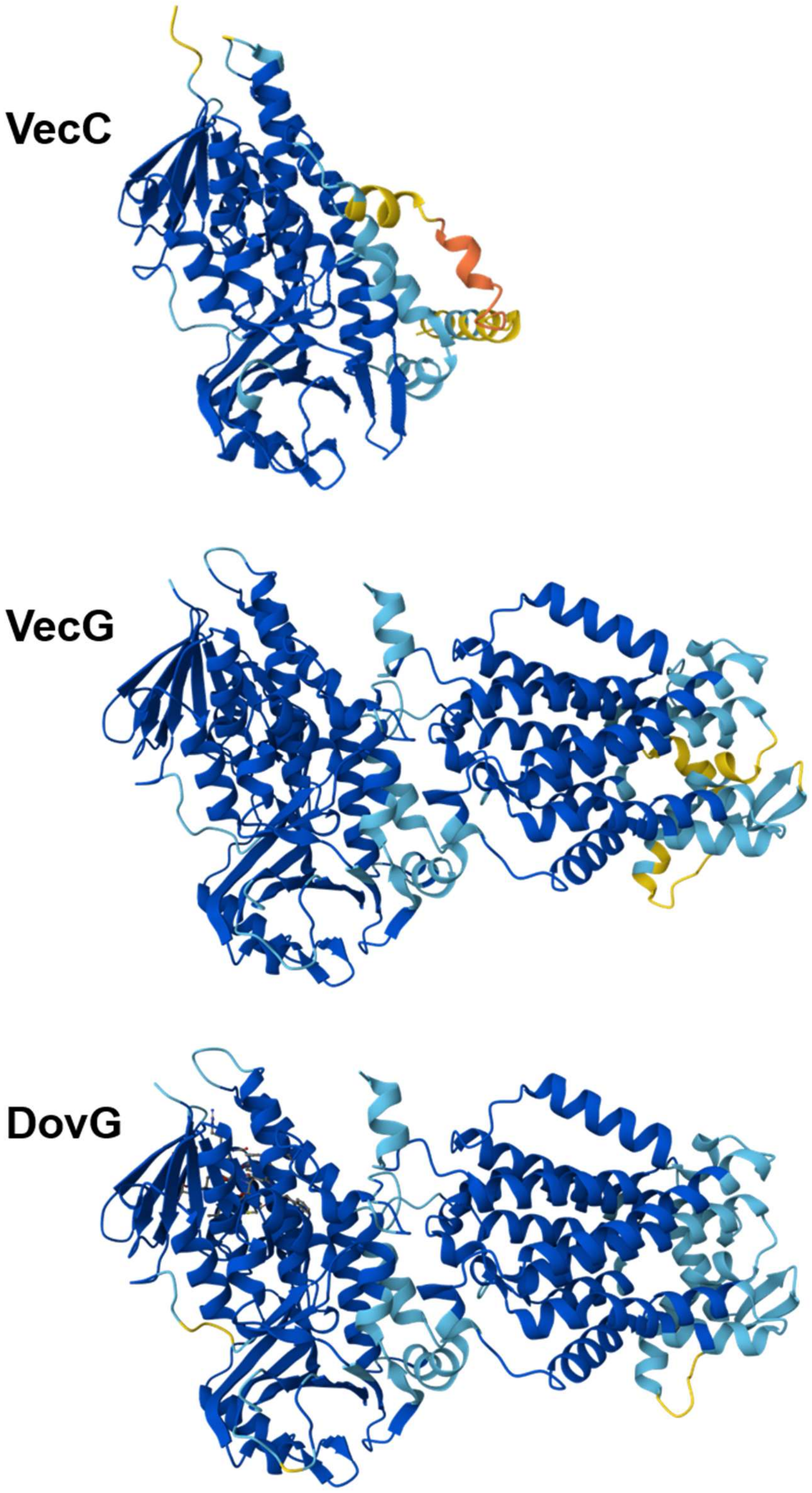

Figure S9. AlphaFold-based structural models of VecC, VecG, and DovG.
The predicted models show the comparable FMO-like fold in all three proteins, with an extended C-terminal helical/transmembrane-associated region in VecG and DovG.

References

1 H.-L. Wei, X.-L. Chen, Y. Dai, L. Yang and S.-M. Li, *Chem. Sci.*, 2025, **16**, 15564.

2 Leo T. Burka, Maya Ganguli, and Benjamin J. Wilson, *J. Chem. Soc., Chem. Commun.*, 1983.

3 R. P. Hodge, C. M. Harris and T. M. Harris, *J. Nat. Prod.*, 1988, **51**, 66.

4 M. Ganguli, L. T. Burka and T. M. Harris, *J. Org. Chem.*, 1984, **49**, 3762.

5 M. R. Green and J. Sambrook, *Molecular cloning: a laboratory manual*, Cold Spring Harbor Laboratory Press, Cold Spring Harbor, New York, 2012, 4th.

6 F. William Studier and Barbara A. Moffatt, *J. Mol. Biol.*, 1986, **189**, 113.

7 C. Bond, Y. Tang and L. Li, *Fungal Genet. Biol.*, 2016, **89**, 52.

8 Y.-M. Chiang, M. Ahuja, C. E. Oakley, R. Entwistle, A. Asokan, C. Zutz, C. C. C. Wang and B. R. Oakley, *Angew. Chem., Int. Ed.*, 2016, **55**, 1662–1665.

9 F. Kindinger, J. Nies, A. Becker, T. Zhu and S.-M. Li, *ACS Chem. Biol.*, 2019, **14**, 1227.

10 J. Fan, G. Liao, F. Kindinger, L. Ludwig-Radtke, W.-B. Yin and S.-M. Li, *J. Am. Chem. Soc.*, 2019, **141**, 4225–4229.

11 X. Xu, R. Huang and W.-B. Yin, *J. Fungi*, 2021, **7**.

12 X. Zhang, T. T. Wang, Q. L. Xu, Y. Xiong, L. Zhang, H. Han, K. Xu, W. J. Guo, Q. Xu, R. X. Tan and H. M. Ge, *Angew. Chem., Int. Ed.*, 2018, **57**, 8184.
